# Composite Anode for PEM Water Electrolyzers: Lowering Iridium Loadings and Reducing Material Costs with a Conductive Additive

**DOI:** 10.1021/acsaem.4c01866

**Published:** 2024-09-06

**Authors:** Kara J. Ferner, Shawn Litster

**Affiliations:** Department of Mechanical Engineering, Carnegie Mellon University, 5000 Forbes Avenue, Pittsburgh, Pennsylvania 15213, United States

**Keywords:** PEM water electrolysis, iridium oxide, low loading, anode catalyst layer, composite anode, conductive additive

## Abstract

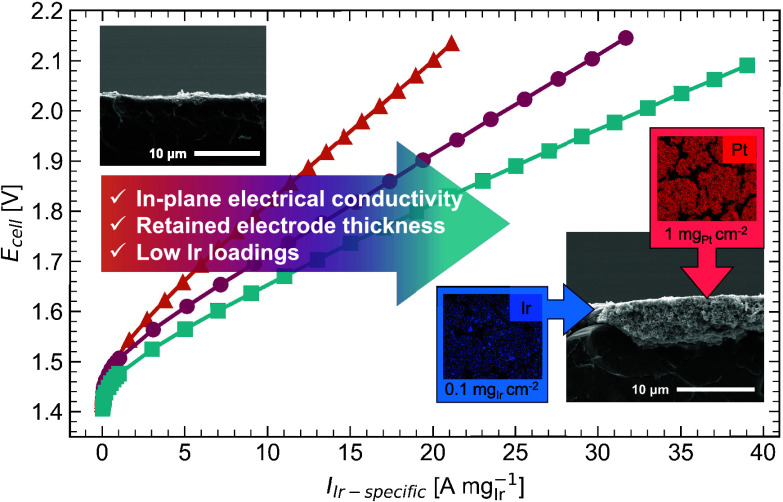

To enable the greater installed capacity of proton exchange membrane water electrolysis (PEMWE) for clean hydrogen production, associated costs must be lowered while achieving high current density performance and durability. Scarce and expensive iridium (Ir) required for the oxygen evolution reaction (OER) is a large contributor to the overall cost, yet high loadings of Ir (1–2 mg_Ir_ cm^–2^) are currently needed in commercial systems to maintain sufficient activity, conductivity, and durability. To meet the aggressive targets for low Ir loadings, we introduce a composite anode approach using a conductive additive that is less expensive than Ir to facilitate robust, high-performance operation with low Ir loading by retaining electrode thickness and in-plane electrical conductivity. In this demonstration, we use platinum (Pt) black as the conductive additive given its high electrical conductivity, acid stability, and current price one-fifth that of Ir. Using a high-activity commercial Ir oxide (IrO*_x_*) catalyst, we present a 95% Ir loading reduction and 80% cost reduction of the anode catalyst materials while maintaining equal current density performance at a cell voltage of 1.8 V. Furthermore, we show enhanced stability of a composite anode compared to an IrO*_x_* anode with loadings of 0.10 mg_Ir_ cm^–2^ via accelerated stress test (AST) and postmortem imaging. With this approach, we show promising results toward lowering Ir loadings and material costs, addressing a significant barrier to the widespread adoption of PEMWE for clean hydrogen production.

## 1 Introduction

Producing clean (zero-carbon-emission) hydrogen will be crucial for addressing the current energy crisis. Hydrogen is a necessary feedstock for industrial processes like steel, cement, and ammonia for fertilizer production; however, most hydrogen produced today is derived from steam methane reforming (SMR), which produces high carbon emissions. Clean hydrogen can displace fossil fuels for these industries, provide renewable energy storage, and power transportation via fuel cell vehicles.^1−3^ Proton exchange membrane water electrolysis (PEMWE) is a promising method for clean hydrogen production due to its high efficiency and operational current density, low-temperature operation, and quick response times.^2−4^ Yet, a significant limitation today is the high cost of materials, particularly of the catalyst needed for the oxygen evolution reaction (OER) at the anode. Iridium oxide (IrO*_x_*) is the most suitable PEMWE anode catalyst material due to its high OER activity and stability in acidic and oxidative environments. Still, its scarcity and cost hinder widespread commercial adoption of PEMWE.^2−6^

For PEMWE installation to ramp up in the coming years, Ir loadings (mg of Ir per cm^2^ of active area) must be reduced. Detailed market analyses by Minke et al.^7^ and Clapp et al.^8^ suggest that given its scarcity, the current commercial Ir loadings near 1–2 mg_Ir_ cm^–2^ will not be sustainable for the GW scale of clean hydrogen production needed to meet long-term decarbonization goals. Widespread efforts are focused on reaching loadings near 0.5–0.1 mg_Ir_ cm^–2^ to maintain PEMWE adoption. For example, the United States Department of Energy (DOE) established a platinum-group-metal (PGM) loading target of 0.125 mg_PGM_ cm^–2^ to be achieved in the next decade.^9^ However, reducing Ir loadings in practice is problematic for two primary reasons: low conductivity and poor durability, mainly due to decreased electrode thickness.

**Figure 1:**
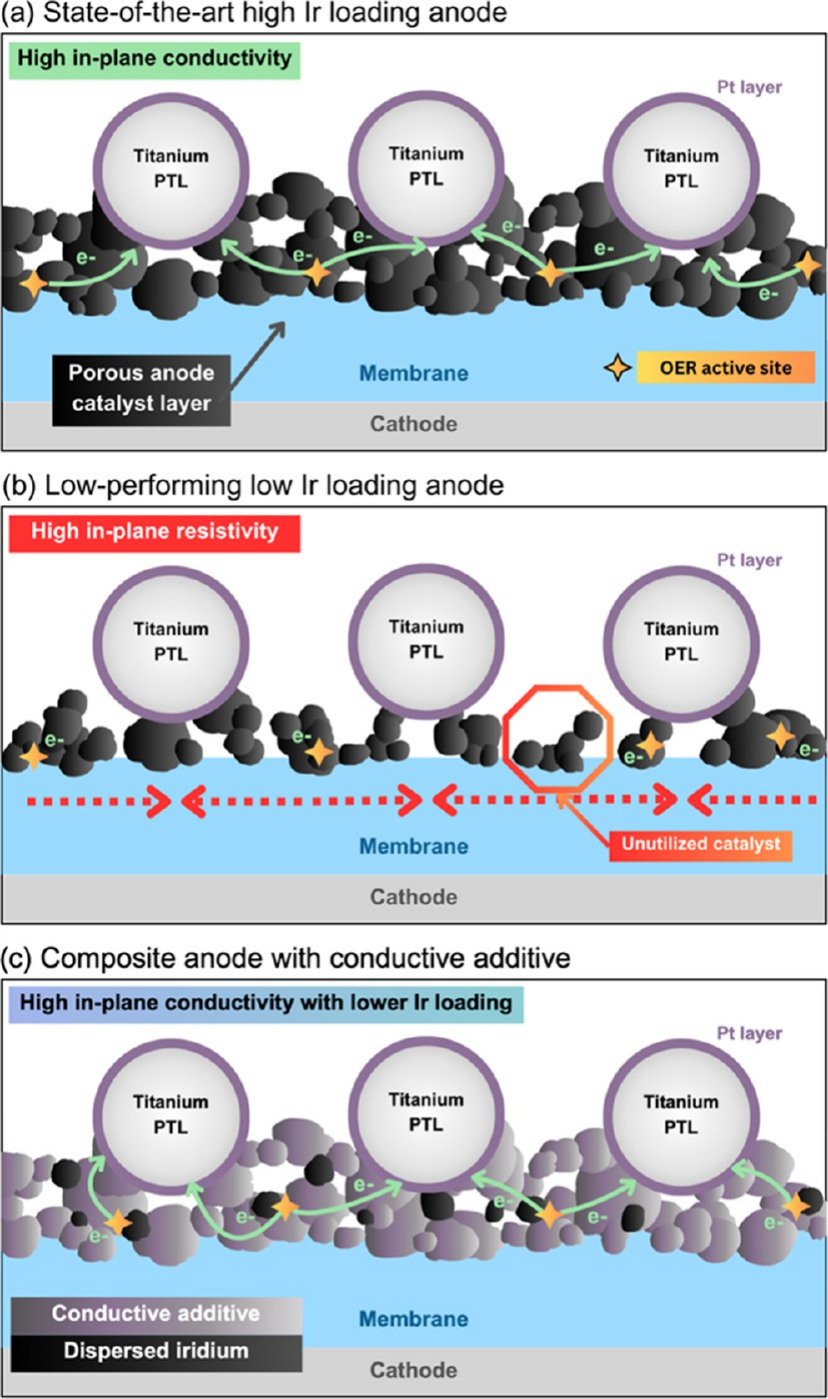
Schematic showing the improved structure using a composite anode with a conductive additive from a cross-sectional view of a PEMWE cell. (a) State-of-the-art high Ir loading PEMWE anode with sufficient thickness and high in-plane conductivity, but the high Ir loading leads to high cost. (b) Typical low-loaded IrO_x_ anode, leading to a thin electrode, electrically disconnected and unutilized catalyst, and low performance. (c) Composite anode using a conductive additive to increase in-plane electrical conductivity and electrode thickness while enabling low Ir loadings.

For typical PEMWE state-of-the-art anode catalyst layers (CLs) that are comprised only of IrO*_x_* and the proton-conducting ionomer, high loadings (1–2 mg_Ir_ cm^–2^) are required to maintain sufficient electrode thickness and conductivity, as shown in Figure 1a. It has been demonstrated that lowering Ir loadings directly correlates to decreased CL thickness,^10,11^ as depicted in Figure 1b, which can result in low inherent conductivity. In recent work by Padgett et al.,^12^ an in-depth analysis of CL resistances determined that the overall resistance in low-loaded anodes is dominated by in-plane electrical resistance rather than ionic resistance or resistance in the through-plane direction. Moreover, extremely thin CLs can suffer from poor connectivity and, in extreme cases, isolated catalyst regions. This can partly be improved by efforts to create more homogeneous CLs with good dispersion of IrO*_x_* particles, such as adjusting catalyst ink properties and rheology^13,14^ and coating parameters and techniques.^15−19^ Taie et al.,^17^ for example, achieved impressive performance with ultralow loadings of less than 0.1 mg_Ir_ cm^–2^ by optimizing their spray coating fabrication, underscoring the importance of homogeneity and careful optimization processes for successful low-loading anodes.

The other impact that CL thinning has on increasing resistances is poor interfacial contact between the CL and the porous transport layer (PTL), which is known to lead to high ohmic loss and decreased electrolyzer efficiency. The PTL, typically made of platinum (Pt)-coated titanium (Ti), is crucial to PEMWE performance as it facilitates gas diffusion and current collection for the anode.^20^ Thus, numerous studies have aimed at optimizing PTL bulk and surface properties for the best tradeoff between good solid contact with the CL and efficient pore phase transport for reactants and products.^21−27^ Moreover, our previous work used nano-CT imaging to find that PTL-CL interactions can result in regions of local CL thinning,^28^ leading to increased resistances. These studies have led to the broad understanding that PTL modifications and the PTL-CL interface are significant in achieving good PEMWE performance, especially for low or ultralow-loaded thin CLs.

Long-term durability is the second major challenge of low Ir loadings for PEMWE anodes. Anodes that are too thin can lack mechanical integrity, leading to performance loss over time via physical breakdown of the CL, Ir dissolution, and consequently decreased electrochemical active surface area (ECSA).^29,30^ Moreover, the nonuniformity that often characterizes thin CLs can facilitate hotspots or areas of high local overpotentials, and this can further accelerate degradation along with the local differences of contact with the PTL.^12,31^ One popular tactic toward maintaining physically robust electrodes with lower Ir loadings is to use support particles or a core–shell design, similar to using Pt catalyst supported on high surface area carbon (Pt/C) for PEM fuel cell cathodes.^32^ While carbon-based materials have poor stability and will readily oxidize in the harsh PEMWE anode conditions, other support particles for IrO*_x_* anodes have been researched, such as TiO_2,_^11,33−36^ TiN,^37,38^ and TiC.^39^ Unfortunately, many of these viable supports that are stable for the PEMWE anode have insufficient conductivity, requiring higher loadings of Ir to form the necessary electrically percolating pathways for high performance. Regmi et al.^40^ addressed this by synthesizing a conductive interfacial layer of Pt between the TiO_2_ support and the Ir catalyst. Furthermore, doping some metal oxides to form supports such as antimony-doped tin oxide (ATO)^41,42^ have been proposed, but there are questions regarding their long-term stability and conductivity. Nonetheless, these works have found that synthesizing IrO*_x_* on support particles is a promising method to increase Ir utilization at low loadings by decreasing Ir packing density while maintaining sufficient electrode thickness.

Other approaches to lower Ir loadings have included creating unique geometries such as nanowires,^43^ nanosheets,^44,45^ nanofiber interlayers,^46^ inverse-opal structures,^47^ or nanostructured thin films (NSTF).^48,49^ Some of these tailored nanostructures, such as the work by Lewinski et al.^48^ to create highly robust NSTF electrodes and the work by Hegge et al.^46^ to create a hybrid IrO*_x_* nanoparticle/nanofiber anode, have shown promising results in both performance and durability while maintaining low Ir loadings. Furthermore, stemming from PTL research, microporous layers (MPLs) or hierarchically graded porous structures for PTLs have also been of recent interest.^50−53^ The use of MPLs effectively increases the spatial contact distribution to the CL, enhancing ohmic and mass transport and distributing mechanical stress across the fragile CL for extended durability. However, one limitation of some of these complex catalyst and electrode structures is that added synthesis steps can increase time and cost, posing challenges for manufacturing scale-up.

### 1.1 Composite Anode with Pt Black Conductive Additive

To address these issues, we present the concept of a composite anode with conductive additive, as depicted in Figure 1c, to enable low Ir loadings while maintaining high current density performance and stability. We hypothesize that incorporating an additive with known high electrical conductivity could first significantly improve the inherent in-plane electrical conductivity of the anode and, second, remove the direct dependence of anode thickness and durability on Ir loading. Owejan et al.^32^ presented a similar approach to lower Pt loadings for PEM fuel cell cathodes by diluting the Pt/C catalyst with additional carbon black. In this work, we investigate a composite anode design for PEMWE anodes by testing Pt black as one option for a conductive additive to dilute the amount of Ir needed. Pt black was chosen as a promising material for this work for several reasons: it is conductive, stable in acid, an existing commercial product at scale, much more abundant than Ir,^7^ and costs approximately 20% that of Ir.^54^

For one, Pt is the current industry standard for coatings of the Ti components in the PEMWE anode (bipolar plates and PTLs). Although Pt oxides can be formed at high anodic potentials (>1.2 V) in acidic media, the benefits of its conductivity and stability have nonetheless proven highly effective in reducing contact resistances and protecting the Ti from passivating into insulating TiO_2._^29,31,36,55,56^ The primary reason Pt has not been used much in the PEMWE anode CL is that it has substantially worse OER activity than Ir, and until 2017, it was more expensive than Ir.^54^ Thus, there was no cost-benefit to using it as a nonactive additive. But notably, the current nominal price of Pt is roughly $1000 oz^–1^, while the current price of Ir is approximately $5000 oz^–1^^54^ While the increasing cost of Ir has added urgency to meeting low-loading targets, it has also motivated research into leveraging Pt as a conductive additive in PEMWE anodes.

Herein, we evaluate the composite anode design using Pt black as the conductive additive. We present results from our in-house fabrication and electrochemical testing of a suite of eight different PEMWE anodes varying in type of anode, type of commercial IrO*_x_* catalyst, and Ir loading. We first present a physical characterization of the anodes’ morphology via scanning electron microscopy (SEM) and energy-dispersive X-ray spectroscopy (EDS), with the characterization of anode sheet resistances via a four-point probe. We then compare electrochemical results across all different anodes with an emphasis on polarization curves, analyzing Ir-specific and price-specific performance to examine reductions in loading and cost. Results from an initial stability screening using a modified accelerated stress test (AST) procedure are also presented. Finally, we discuss key implications and areas of improvement for PEMWE composite anodes.

## 2 Experimental Section

### 2.1 Electrode Fabrication

This work used two types of IrO*_x_* catalyst powders: a hydrous, amorphous type from TKK (TKK TEC77100, Ir 76%, Tanaka Kikinzoku Kogyo K.K., Japan) and a rutile type from Alfa Aesar (AA 043396, Premion, 99.99% (metals basis), min Ir 84.5%, Thermo Fisher Scientific, Waltham, MA). The Pt black powder used for the conductive additive was a high-surface-area type (Alfa Aesar, Platinum black, HiSPEC 1000, Thermo Fisher Scientific, Waltham, MA). Catalyst inks were prepared by first adding the desired amount of catalyst powder (IrO*_x_* and/or Pt black) to a vial, then adding the appropriate amounts of deionized water and 1-proponal to achieve a 1:1 w/w mixture of water to 1-proponal, then adding Nafion D2020 (1000 EW, 20% weight, Ion Power Inc., New Castle, DE) to achieve an ionomer-to-catalyst (I/C) ratio of 0.2. All catalyst inks were ultrasonicated via tip sonication for 10 s at 15% power and ice-bath sonication for 20 min and were then mixed via magnetic stir bar method at 1200 RPM for 30 min (IKA Works, Inc., Wilmington, NC).

CLs were coated onto poly(tetrafluoroethylene) (PTFE) substrates (McMaster Carr, Elmhurst, IL) using an automatic blade gap film coater (MTI Corporation, Richmond, CA). Blade gap values ranged from 50 to 150 μm, with lower blade gaps used to achieve thinner and lower-loaded electrodes. Nitrogen gas was flowed over the coatings for quicker drying times and crack mitigation. After the coatings dried, 5 cm^2^ anode CLs were cut and transferred from the PTFE decal to the membrane via a hydraulic hot press at 130 °C and pressure of 300 psi for 2 min each in four 90° rotations for 8 min total. Loadings were calculated for all electrodes by measuring the gravimetric difference between the 5 cm^2^ decal area before and after transferring the CL and multiplying by the recorded fraction of Ir (and Pt) content in the total solids added to that ink. For the TKK and AA composite anodes and the AA IrO*_x_* anodes, the CLs were decal-transferred onto a Nafion 115 (N115, 127 μm thick) membrane, commercially acquired as one-sided catalyst-coated membranes (CCMs) with 0.1 mg_Pt_ cm^–2^ of Pt/C as the cathode (Ion Power, New Castle, DE). For the TKK IrO*_x_* anodes, the CLs were decal-transferred onto Nafion HP (20 μm) one-sided CCMs with the same 0.1 mg_Pt_ cm^–2^ of Pt/C as the cathode, as this was the available material at the time. An uncoated N115 was hot-pressed to the HP membrane for the TKK IrO*_x_* anodes to achieve similar membrane thicknesses. We do not believe the addition of an HP membrane for TKK IrO*_x_* electrodes, given its small thickness compared to N115, significantly impacted the performance results discussed in the main text. The likely yet minimal impact that the difference in membrane thickness had on high-frequency resistance (HFR) values from electrochemical impedance spectroscopy (EIS) is discussed in the supporting material (Figure S6).

### 2.2 Electrochemical Testing

Membrane electrode assemblies (MEAs) were assembled in electrolyzer cell hardware (Scribner, LLC., Southern Pines, NC) using a carbon-paper gas diffusion layer (GDL) on the cathode side (Sigracet 35 BC carbon paper, Fuel Cell Store, Bryan, TX) and a platinized Ti fiber PTL on the anode side (Bekaert, Zwevegem, Belgium). Bipolar plates had serpentine flow fields and were made of graphite for the cathode and Ti for the anode. Fiberglass PTFE gaskets were used to compress the gas diffusion media between 15 and 20%. The electrolyzer cell was sealed using eight bolts torqued to 100 in-lbf and connected to an in-house electrolyzer testing setup. All electrochemical testing was done using a high-current potentiostat with an 80 A booster (HCP-803, Biologic, Seyssinet-Pariset, France) at 80 °C and ambient pressure. Deionized water was supplied to the anode at a flow rate of 5 mL min^–1^. All cells were conditioned for 90 min with a potential-cycling break-in procedure cycling between 0.6 and 1.6 V in steps of 0.2 V, with 2.5 s holds at 0.6 and 1.6 V and 0.1 s holds during intermediate steps. During conditioning, the cathode was supplied with humidified N_2_ gas at a flow rate of 200 sccm. After conditioning, cyclic voltammetry was performed for 11 cycles at 50 mV/s, with humidified hydrogen gas supplied to the cathode at a flow rate of 200 sccm. Polarization curves were then recorded in galvanostatic mode, with no flow provided to the cathode. Each current step was held for 2 min, recording measurements every 100 ms. Data points for polarization curves were calculated as an average of the last 100 measurements at each current step.

### 2.3 Stability Testing

Stability testing was conducted for the AA composite and IrO*_x_* anodes after the standard beginning-of-test (BOT) performance characterization at 80 °C, ambient pressure, and with 5 mL min^–1^ of deionized water supplied to the anode. The cells were cycled between 1.45 and 2 V with a square-wave profile and cycle time of 10 s for 5.8k cycles a day (15 h of active cycling time). Every 360 cycles (1 h), the cell was taken to a galvanostatic hold at 2 A cm^–2^ for 1 min and was followed by a potentiostatic EIS measurement at 1.45 V at frequencies from 100 kHz to 100 mHz with 5 mV amplitude, then immediately returned to cycling. After the 5.8k cycles, a polarization curve was taken, and then the cell was purged with nitrogen, shut down, and returned to the next day. This protocol was repeated for another 5.8k cycles to reach 10.8k cycles (30 h).

### 2.4 Electrode Physical Characterization

#### 2.4.1 Sheet Resistance via Four-Point Probe

Electrical sheet resistances of the anodes were measured using a four-point probe (302 Resistivity Stand with SP4 probe head, Lucas Signatone Corp., Gilroy, CA). The probe tips had a radius of 0.0016 in. and a spacing of 0.04 in. The probe tip material was tungsten carbide, and the spring pressure was 85 g. Measurements were taken for at least one sample from each type of CL and on 5 cm^2^ areas of the coatings on the PTFE decal. These portions of CL differed from those used for electrochemical testing because the four-point probe damages the CL after contact. We ensured the requirements were met for accurate four-point probe measurements: the edge of the sample was at least ten times the spacing from the nearest probe tip, and the thickness of the sample was less than 0.4 times the spacing between probe tips, which in this case would be less than ∼400 μm. Using a benchtop power supply (Keithley Instruments, Inc., Cleveland, OH) connected to the four-point probe, a small current was supplied until the voltage measured between 1 and 100 mV, and measurements were recorded in 15 locations for each CL. Sheet resistance, *R*_s_, was calculated in units of Ω·sq^–1^, using the equation based on four-point probe theory, with current source value, *I*, and the average of the voltage measurements, *V*, in eq 1
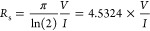
1If the sample’s thickness, *d*, is known, one could calculate the film’s resistivity, ρ, and conductivity, σ, using eqs 2 and 3.
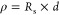
2
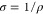
3

However, due to the nonuniformity of CL thicknesses in this work, we report sheet resistance values rather than bulk resistivity or conductivity, which depend on accurately measuring the sample thickness.

#### 2.4.2 Scanning Electron Microscopy (SEM)

Cross-sectional SEM images were obtained using freeze-fracturing. Samples were submerged in liquid nitrogen and fractured to reveal clean cross-sections. Due to the destructive nature of this sample preparation, separate CL regions were cut from the PTFE decals near where the CLs used for electrochemical testing were taken. These regions were hot-pressed onto N115 for freeze-fracturing and loading measurement, and the loadings for these imaged regions are specified in the text. After freeze-fracturing, the exposed cross-sections of each sample were mounted onto tilted SEM stubs, sputtered with 2 nm of gold for conductivity, and imaged at a working distance of 10 μm using an Everhardt Thornley Detector (ETD), an accelerating voltage of 10 kV, and a spot size of 3.0 (FEI Quanta 600 FEG SEM, Thermo Fisher Scientific, Waltham, MA).

#### 2.4.3 Energy Dispersive X-ray Spectroscopy (EDS)

EDS mapping was acquired for surface images of CL samples (on N115) at an accelerating voltage of 20 kV and spot size of 5.0, using Oxford Instruments’ AzTec software (Oxford INCA EDS System full analytical, XMAX 80 mm SDD EDX detector).

## 3 Results and Discussion

Table 1 outlines the eight different anodes tested in this study. We tested two commercial IrO*_x_* catalysts, one from TKK and one from Alfa Aesar (AA). We fabricated composite anodes using these IrO*_x_* catalysts at a loading of 0.10 mg_Ir_ cm^–2^ and with Pt black conductive additive. We compared them to IrO*_x_* anodes at varying Ir loadings. As a control experiment, we also evaluated an anode containing only Pt black and no IrO_x_.

**Table 1:** Summary of the Eight Different Anodes Used for Electrochemical Testing in This Work^a^

type of anode	commercial IrO*_x_* used	Ir loading (mg_Ir_ cm^–2^)	Pt loading (mg_Pt_ cm^–2^)
IrO*_x_*	TKK	1.88	
IrO*_x_*	TKK	0.51	
IrO*_x_*	TKK	0.24	
composite	TKK	0.10	1.21
IrO*_x_*	AA	0.18	
IrO*_x_*	AA	0.10	
composite	AA	0.10	1.05
Pt black only			0.98

a The two commercial IrO*_x_* catalysts were acquired from TKK and Alfa Aesar (AA; Thermo Fisher Scientific). One anode containing only Pt black was tested for a control experiment.

### 3.1 Physical Characterization

**Figure 2:**
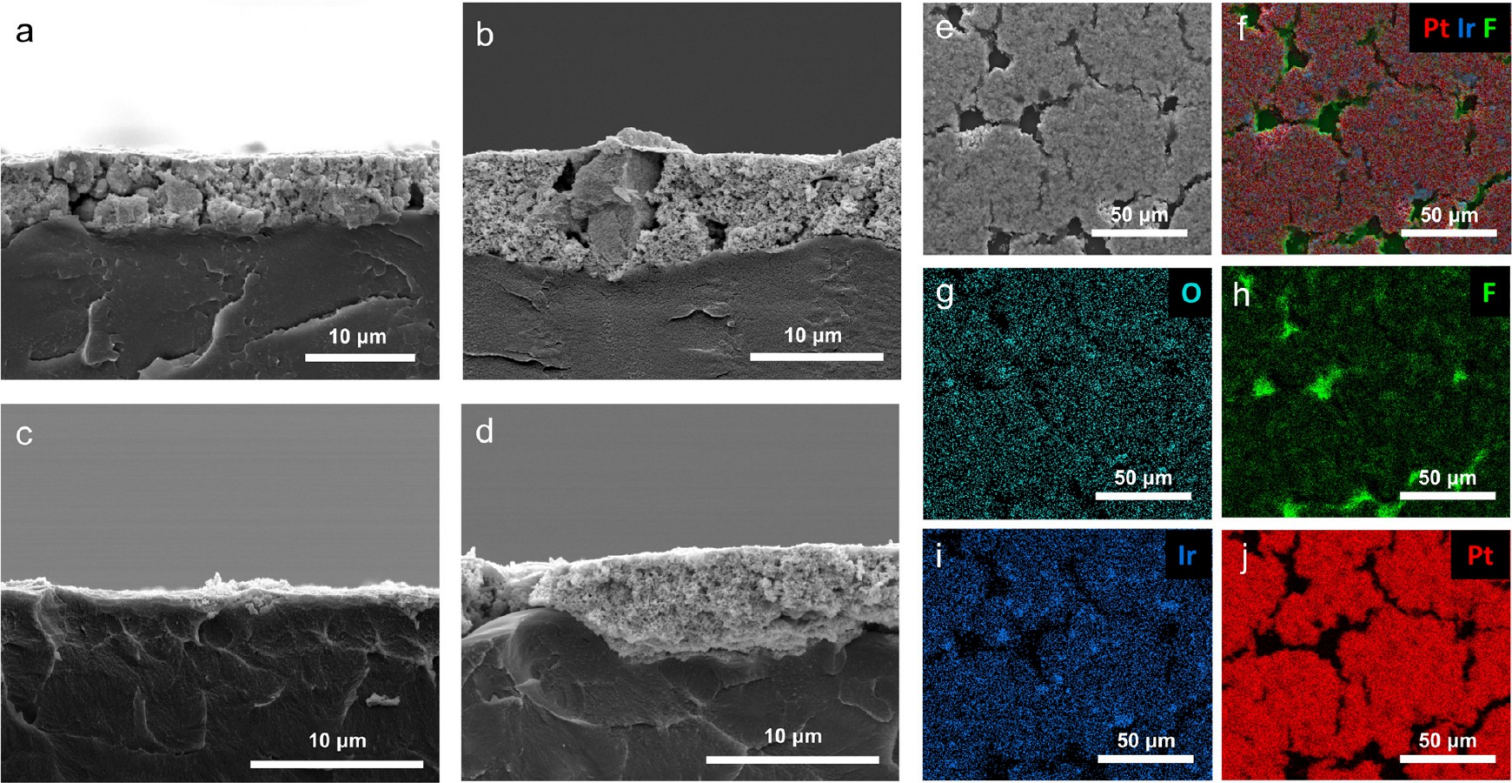
Cross-sectional SEM images of (a) TKK IrO*_x_* anode with medium loading (0.51 mg_Ir_ cm^–2^), (b) TKK composite anode comprised of 0.10 mg_Ir_ cm^–2^ and 1.21 mg_Pt_ cm^–2^, (c) AA IrO*_x_* anode with low loading (0.11 mg_Ir_ cm^–2^), and (d) AA composite anode comprised of 0.12 mg_Ir_ cm^–2^ and 1.26 mg_Pt_ cm^–2^. (e) Surface SEM image of the AA composite anode with (f) EDS elemental mapping showing the distribution of (g–j) O, F, Ir, and Pt. All images show the CL on N115 after decal transfer via hot-pressing. SEM samples were acquired from the same coatings but in regions adjacent to those used for electrochemical testing, resulting in some slight loading variations.

To first investigate electrode morphology and thickness, we characterized the anodes using cross-sectional SEM imaging. Figure 2a shows a cross-sectional SEM image of an IrO*_x_* anode using the TKK catalyst with a loading of 0.51 mg_Ir_ cm^–2^, and Figure 2b shows a cross-sectional SEM image of the composite anode using the same TKK catalyst with loadings of 0.10 mg_Ir_ cm^–2^ and 1.21 mg_Pt_ cm^–2^. Figure 2c shows an IrO*_x_* anode using the AA catalyst with a lower loading of 0.11 mg_Ir_ cm^–2^, and Figure 2d shows the AA composite anode with loadings of 0.12 mg_Ir_ cm^–2^ and 1.26 mg_Pt_ cm^–2^. Most noticeably, the cross-sectional images of both TKK anodes (IrO_x_ and composite) show large solid particles, indicating they are characteristic of the as-received commercial IrO*_x_* catalyst powder. The surface SEM images of the TKK composite anode (Figure S1) also revealed instances of these large particles. Meanwhile, neither AA anode contained similar large, outlier IrO*_x_* particles in the cross-sectional or surface images (Figure S2), indicating that the AA catalyst provided smaller and more uniform particle sizes.

Regarding the impact on electrode thickness, the region of the TKK composite anode in Figure 2b shows a similar thickness as the IrO*_x_* anode in Figure 2a with an 80% reduced Ir loading (0.10 compared to 0.51 mg_Ir_ cm^–2^). Figure 2c shows that at 0.11 mg_Ir_ cm^–2^, the AA IrO*_x_* anode without Pt black conductive additive was extremely thin. In contrast, the AA composite anode had significantly improved electrode thickness for approximately the same loading. While both composite anodes generally aided in retained or improved thickness for low Ir loadings, local variations are also visible (see Figure S3 for additional cross-sectional images for all samples). While local thickness variations and nonuniformities are common for CLs fabricated via blade-coating techniques,^28^ this highlights an area for further improvement of composite anode fabrication.

Figure 2e–j show EDS surface mapping of oxygen (O), fluorine (F), Ir, and Pt signals from the AA composite anode (0.12 mg_Ir_ cm^–2^ and 1.26 mg_Pt_ cm^–2^) decal-transferred onto N115 membrane. This EDS mapping first confirms that the AA IrO*_x_* particles dispersed well throughout a robust matrix of Pt black particles. Some regions of slightly stronger Ir signal indicate possible aggregation. The F signal throughout the CL can be attributed to the Nafion perfluorosulfonic acid (PFSA) ionomer dispersion added in catalyst ink formulation; however, the brightest F signals were due to the exposed N115 membrane visible through small cracks, which were consistent in the TKK composite anode as well (Figure S4). Additional EDS images showing a larger field of view are also found in Figures S4 and S5 for the TKK and AA composite anodes, respectively. These nonuniformities of thickness variations and small cracks suggest that longer-term optimization of the fabrication and coating of these anodes could yield additional performance and durability increases. The optimized variables could include the deposition process, catalyst particle and pore size distributions, ionomer content, solvents, and catalyst-to-additive ratio. Nonetheless, for both types of catalyst, the Pt black particle morphology mixed well with the IrO*_x_* particles and allowed for improvement in CL thickness and connectivity at low Ir loadings.

**Figure 3:**
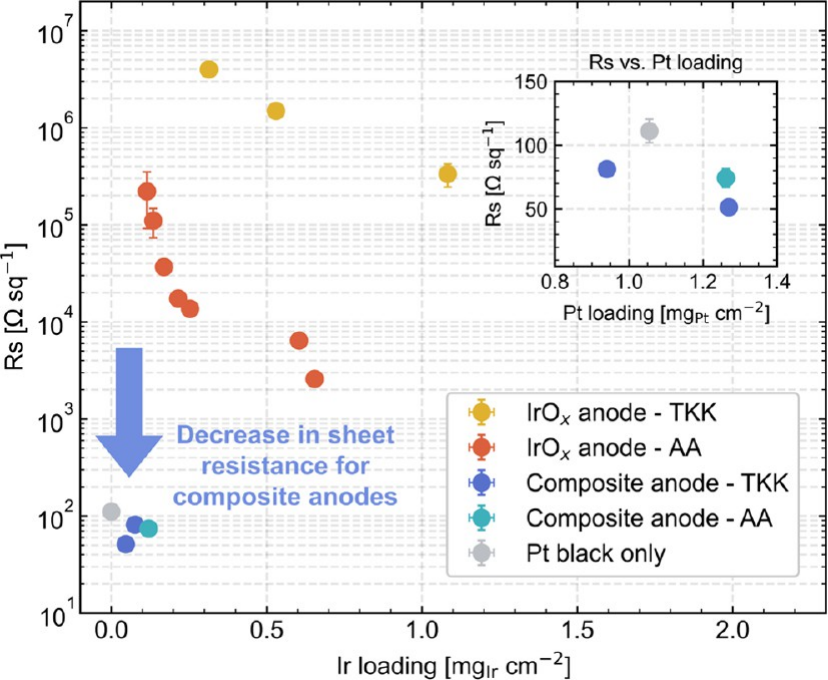
A semilogarithmic plot of sheet resistance (*R*_s_) measured by a four-point probe vs Ir loading for different IrO*_x_* anodes, composite anodes, and an anode containing only Pt black. The plot inset shows sheet resistance vs Pt loading for composite anode samples and one Pt black anode. All data points represent an average of 15 measurements within a 5 cm^2^ area, with error bars representing one standard deviation.

Sheet resistances (*R*_s_) were then characterized via a four-point probe to confirm that the Pt black conductive additive substantially improved in-plane electrical conductivity. Sheet resistance values provided insight into changes in anode material conductivity without requiring precise anode thickness measurements, which can be difficult to extract as a single value given the local variations in the thickness of these anodes. Figure 3 shows the sheet resistance measurements (Ω·sq^–1^) vs Ir loading, and the inset shows sheet resistance vs Pt loading for the composite anodes and an anode containing only Pt black. For both commercial catalysts, IrO*_x_* anodes show increased sheet resistance with decreased Ir loadings, indicating that in-plane ohmic losses become more severe at low Ir loadings. For IrO*_x_* anodes, the AA catalyst was more conductive than the TKK catalyst, with approximately 2 orders of magnitude lower sheet resistance for a given Ir loading. The anode with only Pt black had a sheet resistance of ∼100 Ω·sq^–1^, and the composite anodes with Pt black conductive additive showed slightly lower sheet resistance values in the range of ∼50–100 Ω·sq^–1^. This confirmed that the Pt black was a highly conductive material on its own and that, when combined with a small loading of Ir, yielded a decrease in sheet resistance of three to 5 orders of magnitude (depending on commercial catalyst type) compared to IrO*_x_* anodes with similar Ir loadings. We concluded from these results that providing a sufficient, percolating quantity of conductive additive to the anode can dramatically increase the in-plane electrical conductivity throughout the CL.

### 3.2 Electrochemical Performance

#### 3.2.1 Polarization Curves

**Figure 4:**
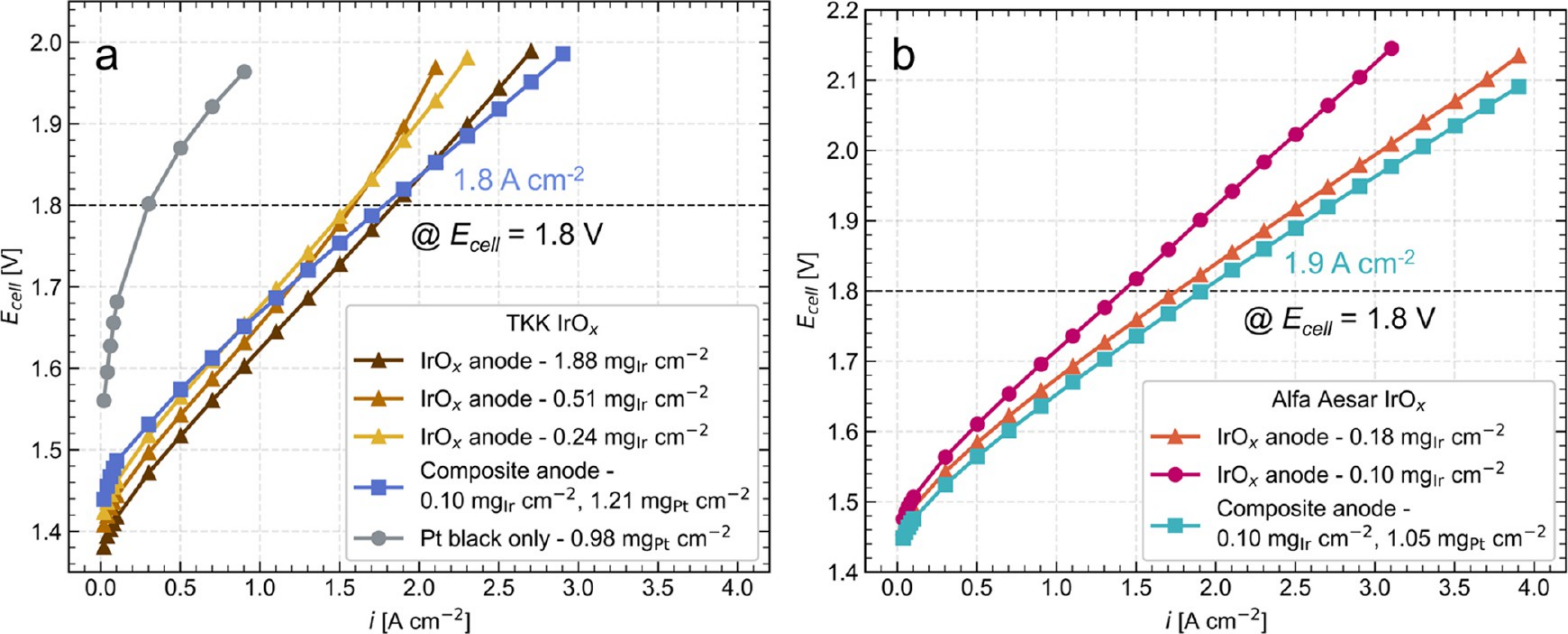
Polarization curves for (a) TKK anodes and (b) AA anodes, showing cell voltage, *E*_cell_, as a function of geometric active area normalized current density in A cm^–2^.

Following physical characterization, we evaluated the electrochemical performance of the composite anodes against IrO_x_ anodes of the same commercial catalyst with varying Ir loadings. Figure 4a shows the polarization curves for the TKK catalyst, comparing three IrO*_x_* anodes containing a high, medium, and low Ir loading (1.88, 0.51, and 0.24 mg_Ir_ cm^–2^, respectively) to a composite anode with 0.10 mg_Ir_ cm^–2^ and 1.21 mg_Pt_ cm^–2^. For the IrO*_x_* anodes, performance generally scaled inversely with Ir loading; however, the composite anode showed polarization enhancement for a lower Ir loading. At current densities higher than 1.0 A cm^–2^, the composite anode showed improved overpotential compared to the 0.24 mg_Ir_ cm^–2^ IrO*_x_* anode. At a typical cell operating voltage of 1.8 V, the high-loaded IrO*_x_* anode and the composite anode had approximately the same operating current density of 1.8 A cm^–2^. This represents a drastic 95% decrease in Ir loading, from 1.88 to 0.10 mg_Ir_ cm^–2^, while maintaining similar performance at 1.8 V, enabled by adding Pt black as a conductive additive at a loading of 1.21 mg_Pt_ cm^–2^. The composite anode’s performance advantages above 2 A cm^–2^ are likely a result of the improved in-plane electrical conductivity, enabling a lower rate of increasing overpotential caused by ohmic losses.

Figure 4a also shows that an anode containing only Pt black at a loading of 0.98 mg_Pt_ cm^–2^ performed poorly, reaching a cell voltage of 1.8 V at ∼300 mA cm^–2^. The cell voltage of the Pt black anode rapidly increased at lower current densities, and adding a large amount of Pt black in the composite anode did not improve its low current performance, indicating negligible kinetic benefit for OER when used with the TKK IrO*_x_*. EIS results (Figure S6) also agreed, showing that the Pt black anode had a lower high-frequency resistance (HFR) yet a much larger charge transfer resistance than the TKK composite anode. These results, along with the low sheet resistance measurements, confirmed that adding Pt black increased the performance of the composite anode design via improvements in ohmic effects rather than kinetic effects.

Figure 4b shows the polarization curve comparison for the AA IrO*_x_* anodes (0.18 and 0.10 mg_Ir_ cm^–2^) and the AA composite anode with 0.10 mg_Ir_ cm^–2^ and 1.05 mg_Pt_ cm^–2^. These measurements first showed that the composite anode significantly outperformed an IrO*_x_* anode at the same loading of 0.10 mg_Ir_ cm^–2^. Adding 1.05 mg_Pt_ cm^–2^ to the composite anode resulted in a significant ∼100 mV reduction in overpotential at 2 A cm^–2^ while maintaining low Ir loading. The AA composite anode also slightly outperformed an IrO*_x_* anode with a higher loading of 0.18 mg_Ir_ cm^–2^. Moreover, the AA composite anode held a current density of about 1.9 A cm^–2^ at 1.8 V, slightly better than the TKK composite anode with 1.8 A cm^–2^ at 1.8 V. This difference in performance between the two IrO*_x_* catalyst types (TKK vs AA) indicates that the impact of the Pt black depends on the catalyst. The precise correlations between the effectiveness of the Pt black additive and the structural and chemical differences in catalyst type should be investigated via advanced characterization techniques in future work.

#### 3.2.2 Ir-Specific Performance

**Figure 5:**
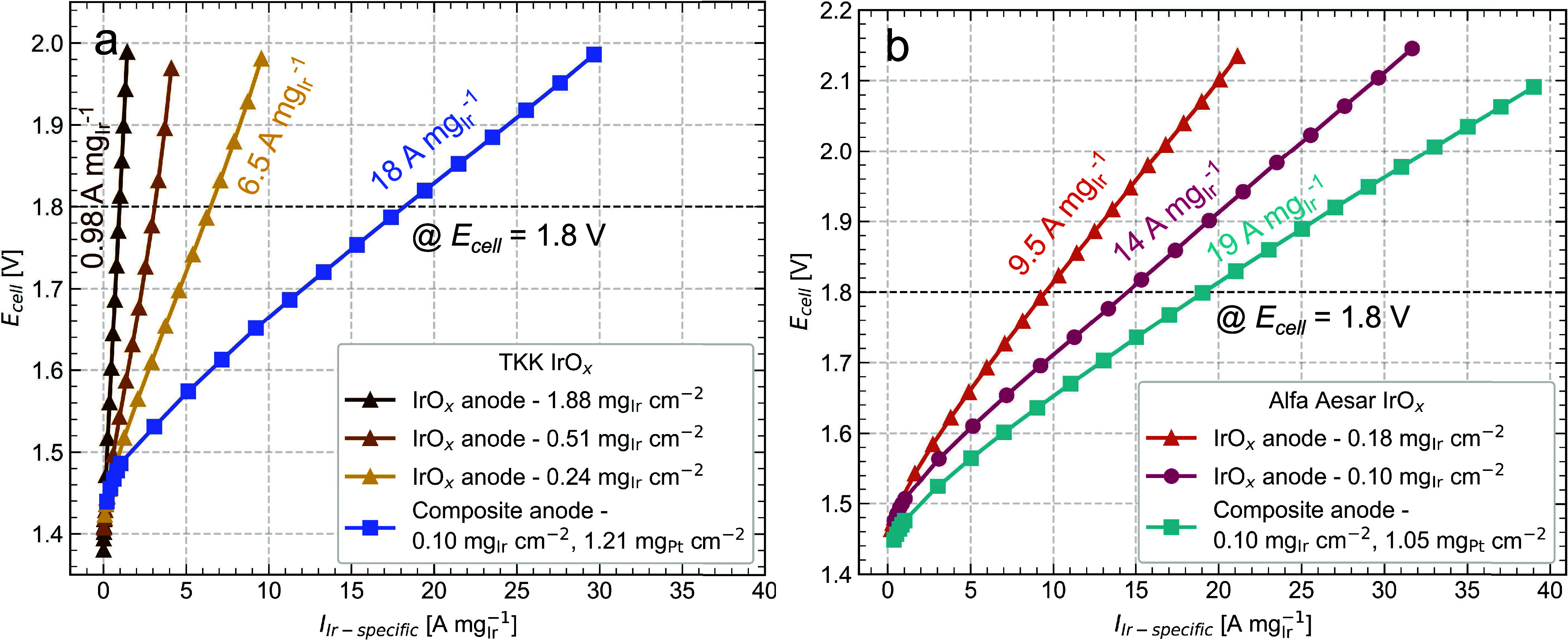
Polarization curves for (a) TKK anodes and (b) AA anodes, showing cell voltage, *E*_cell_, as a function of Ir-specific current in A mg_Ir_^–1^, which is the current normalized to the Ir loading.

To further highlight the composite anodes’ performance benefit at low Ir loadings, we present the polarization curve measurements normalized by Ir loading to show the Ir-specific current. For TKK anodes, Figure 5a shows a 2.8-fold increase in Ir-specific current at 1.8 V for the composite anode (18 A mg_Ir_^–1^) compared to the 0.24 mg_Ir_ cm^–2^ IrO*_x_* anode (6.5 A mg_Ir_^–1^) and an 18-fold increase compared to the 1.88 mg_Ir_ cm^–2^ IrO*_x_* anode (0.98 A mg_Ir_^–1^). Figure 5b shows that the composite anode also improved the Ir-specific performance for AA anodes from 14 to 19 A mg_Ir_^–1^ while keeping an Ir loading of 0.10 mg_Ir_ cm^–2^. Interestingly, across all IrO*_x_* anodes, regardless of catalyst supplier, the Ir-specific performance increased with decreasing loading, with the best performance measured for the AA low-loaded 0.10 mg_Ir_ cm^–2^ anode at 14 A mg_Ir_^–1^. This indicates that some Ir is not effectively utilized at higher loadings, as noted by others.^17^ This would suggest that Ir-specific current performance can be improved simply by lowering the Ir loading; however, the lowest achievable loading before significant performance losses takeover and durability is compromised is unclear and dependent on a variety of factors such as the anode configuration, PTL properties, membrane thickness, and operating conditions. Nonetheless, these results show that using a conductive additive in the anode can improve the current performance at a given low Ir loading.

The current density performance demonstrated with both composite anodes is highly comparable to other research on unique anode designs aimed at lowering Ir loadings. Hegge et al.’s approach of an IrO*_x_* nanoparticle/nanofiber hybrid anode performed similarly: 1.86 A cm^–2^ at 1.8 V with a slightly higher loading of 0.2 mg_Ir_ cm^–2^.^46^ Chatterjee et al. also showed similar current density at 1.8 V with nanoporous-Ir nanosheets but at an ultralow-loading of 0.06 mg_Ir_ cm^–2^,^45^ and other studies on novel CL structures or supported catalysts display promising performance and activity.^44,47,57,58^ Alternatively, this composite anode design has an advantageously straightforward fabrication process. Furthermore, Taie et al.’s work achieved impressive performance of 1.80 and 1.39 A cm^–2^ at 1.8 V for ultralow loadings of 0.039 and 0.011 mg_Ir_ cm^–2^, respectively, via spray coating optimization.^17^ With such low Ir content, this translated to a maximum Ir-specific current performance of ∼130 A mg_Ir_^–1^ of the 0.011 mg_Ir_ cm^–2^ anode, which suggests that further reductions in Ir for our composite anode approach may be possible with improved fabrication for homogeneity and highlights the need for further investigation into catalyst/additive interactions.

#### 3.2.3 Price-Specific Performance

**Figure 6:**
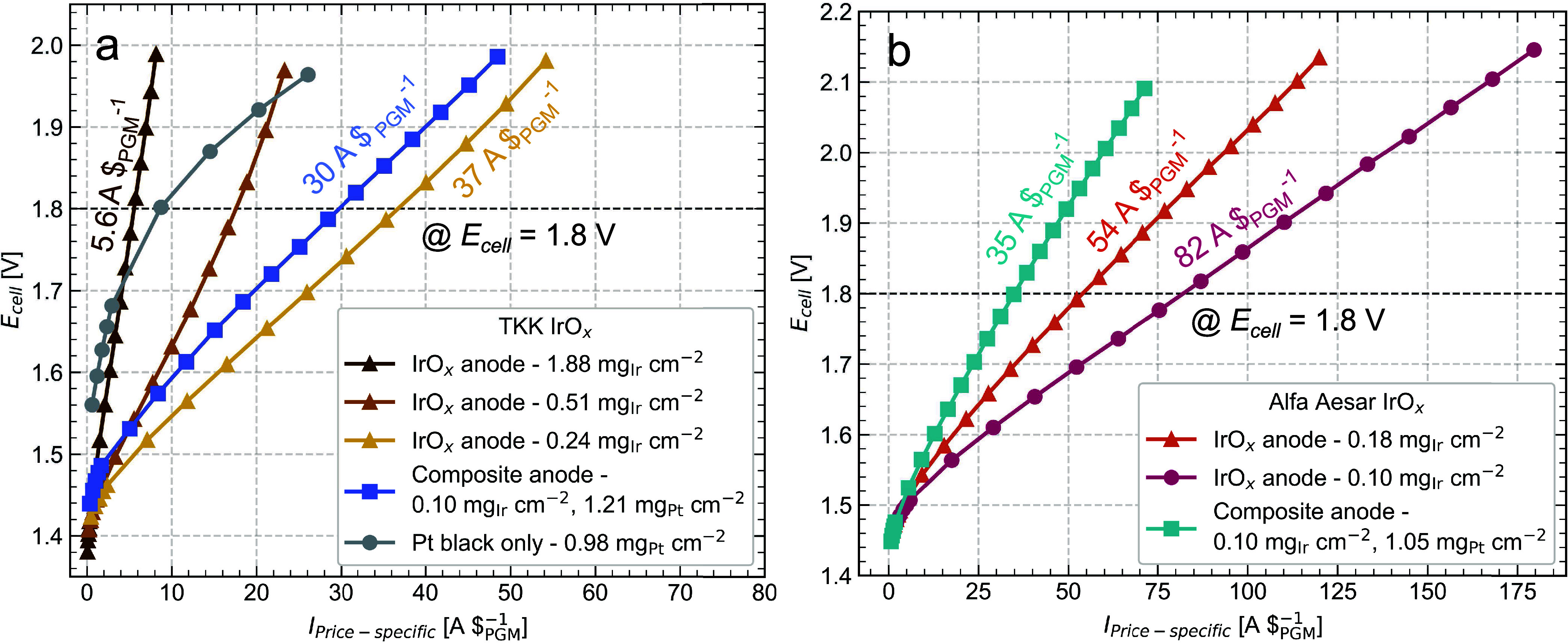
Polarization curves for (a) TKK anodes and (b) AA anodes, showing cell voltage, *E*_cell_, as a function of price-specific current in A $_PGM_^–1^, which is the current normalized to the total cost of PGMs in the anode (Ir + Pt).

To put these results in the context of overall cost, we also present the polarization curve performance normalized by the total cost of PGMs (Ir + Pt) in the anode (price-specific current), assuming nominal prices of $5000 oz^–1^ for Ir and $1000 oz^–1^ for Pt. Figure 6a shows that for the TKK catalyst, the composite anode had a 5-fold increase in A $_PGM_^–1^ at 1.8 V (or 80% cost reduction in $_PGM_ A^–1^) compared to the high-loaded IrO*_x_* anode at 1.88 mg_Ir_ cm^–2^, which is in the typical range of 1–2 mg_Ir_ cm^–2^ loadings for current commercial applications. This result is particularly promising in the context of PEMWE scale-up. As PEM electrolyzer plants increase in size and power capacity, the cost of raw PGM materials becomes a more significant portion of the total cost compared to other expenses subject to the benefits of economies of scale (i.e., electricity costs and balance of plant).^2,59^ Thus, decreasing the associated cost of the raw PGM materials will be even more critical as greater capacity electrolyzer plants are realized.

In contrast, the lower-loaded IrO*_x_* anode using the TKK catalyst had a better price-specific current performance (37 A $_PGM_^–1^) than the composite anode (30 A $_PGM_^–1^), shown in Figure 6a. Similarly, with the AA catalyst, Figure 6b shows the two IrO*_x_* anodes had better price-specific current than the composite anode. The reason for the composite anodes’ lower price-specific current despite their significant improvement in Ir-specific current (Figure 5) is the use of relatively high Pt loadings. Although less expensive than Ir, Pt is still costly compared to more abundant, non-PGM materials. Therefore, the relatively high Pt loadings (>1 mg_Pt_ cm^–2^) in these composite anodes suggest that efforts toward lower Pt loadings could further improve price-specific performance. Moreover, Pt and other PGMs are subject to highly dynamic supply and demand chains, which can result in unstable prices with time.^7,54^ Considering these factors, a cheaper and more abundant conductive additive would be a better material selection; however, the requirements of acid stability and resistance to oxidation at the high anodic potentials are significant limitations to the range of possible materials for the PEMWE anode.

To briefly address the interesting result in Figure 6a that at cell voltages approaching 2 V, the Pt black anode outperformed the two highest loading IrO*_x_* anodes in A $_PGM_^–1^, we refer to Figure 4a to recall that the current density performance in A cm^–2^ of the Pt black anode was significantly worse at any acceptable operating cell voltages. Practically, the much lower operating current density of an anode containing only Pt black and no IrO*_x_* catalyst would require multiple PEMWE cells. High additional costs for the scale-up of those stacks would be incurred, negating improvements in the price-specific performance related to the anode CL.

### 3.3 Stability

#### 3.3.1 Electrochemical Performance Degradation

In addition to high performance, maintaining stability and durability with low Ir loadings is essential for commercially relevant PEMWE anodes. Performance loss over time can occur via several degradation mechanisms, and the type and severity of the degradation depends on cell operating conditions, catalyst material, and loading, among other factors.^30,58,60,61^ In this work, our primary goal was to perform an initial catalyst stability screening to evaluate possible adverse reactions between the Pt black additive and IrO*_x_* catalyst in the composite anode. Thus, we used a voltage cycling AST procedure with a square wave profile between 1.45 and 2 V, modified from the previous work of Alia et al.^30^ To maximize possible catalyst degradation effects and shorten the experiment duration for this stability screening, we used 10-s cycles (10.8k total AST cycles in 30 h for each sample). We conducted the AST on the three AA samples since the AA IrO_x_ catalyst is more widely used in the field and has shown acceptable stability in previous work.^12,62^ We also note that the TKK IrO*_x_* did not demonstrate acceptable stability at higher voltages >2 V in our initial testing and was thus deemed a less appropriate choice of catalyst to effectively evaluate the impact of the composite anode approach on stability.

**Figure 7:**
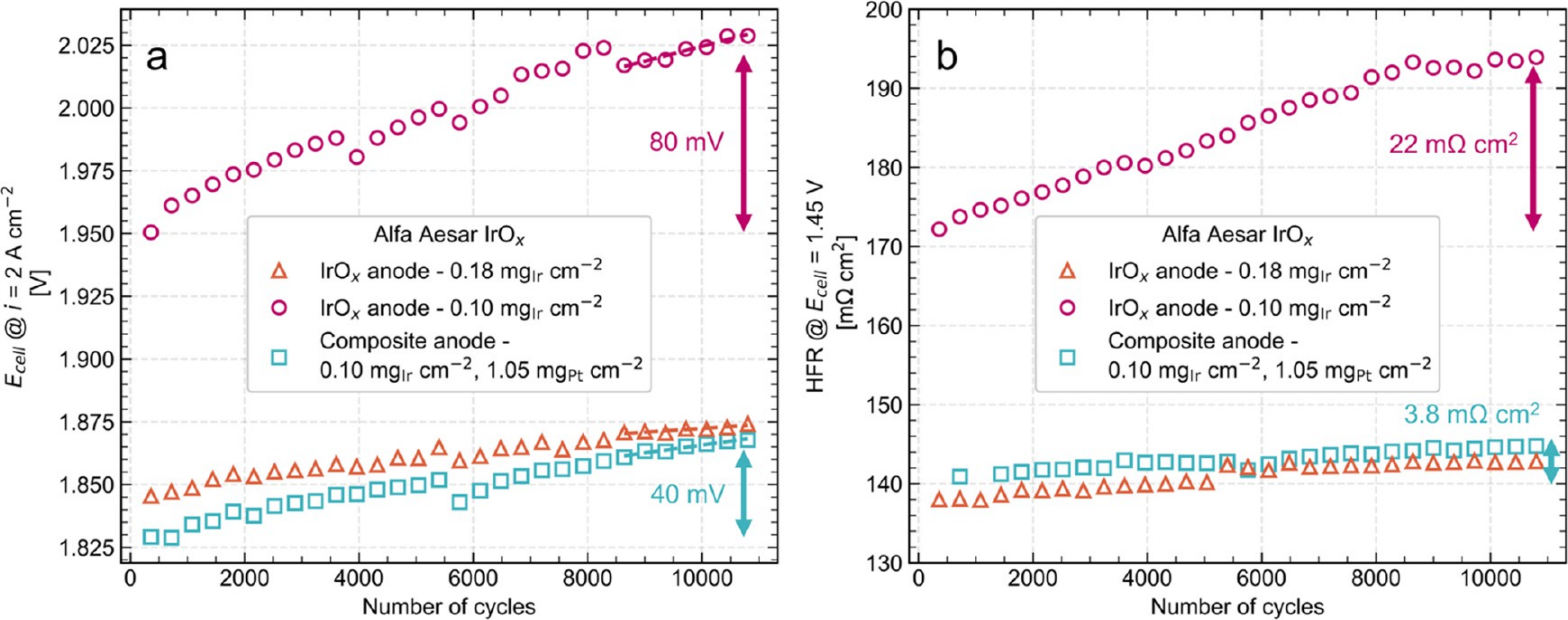
Stability results from AST voltage cycling of AA anodes, showing (a) the change in cell voltage at 2 A cm^–2^ and (b) the change in HFR at 1.45 V as a function of the number of AST cycles. We used square wave voltage cycling between 1.45 and 2 V with a cycle time of 10 s and completed 10.8k cycles for each sample. Each data point represents a measurement acquired every 360 cycles (1 h), where the cell was held for 1 min at 2 A cm^–2^ followed by potentiostatic EIS at 1.45 V with frequencies from 100 kHz to 100 mHz.

Figure 7a,b show the changes in cell voltage at 2 A cm^–2^ and HFR at 1.45 V, respectively, vs the number of AST cycles for the three AA anodes. Figure 7a shows that the cell voltage increase was most significant for the 0.10 mg_Ir_ cm^–2^ IrO*_x_* anode, with an 80 mV overpotential increase from the beginning to the end of the AST. In contrast, the composite anode showed only a 40 mV overpotential increase. Since both anodes contained the same Ir loading, this result indicates that the composite anode reduced the 2 A cm^–2^ cell voltage increase by 50%, effectively enhancing stability. Polarization curves measured after 5.4k and 10.8k AST cycles (Figure S8) also confirm that the composite anode reduced performance degradation comparatively at a loading of 0.10 mg_Ir_ cm^–2^.

Figure 7b shows that the initial HFR value was significantly higher for the 0.10 mg_Ir_ cm^–2^ IrO*_x_* anode at 172 mΩ·cm^2^ compared to 138 and 141 mΩ·cm^2^ for the 0.18 mg_Ir_ cm^–2^ IrO*_x_* anode and the composite anode, respectively. This indicates larger BOT ohmic losses for the thin, 0.10 mg_Ir_ cm^–2^ IrO*_x_* anode and shows that the Pt black conductive additive reduced the BOT HFR for low Ir loadings. This result is consistent with the decreased sheet resistance of composite anodes shown in Figure 3, and it explains a portion of the larger overpotentials for the low-loaded 0.10 mg_Ir_ cm^–2^ IrO*_x_* anode shown in the BOT polarization curves in Figure 4b. Figure 7b also reveals that the 0.10 mg_Ir_ cm^–2^ IrO*_x_* anode had the most significant increase in HFR from beginning to end of the AST of 22 mΩ·cm^2^, while the composite anode HFR increased by less than 4 mΩ·cm^2^ and the 0.18 mg_Ir_ cm^–2^ IrO*_x_* anode HFR increased by 5 mΩ·cm^2^. The total impedance spectra (from which the HFR values were calculated) for all three samples can be found in Figure S9, which also showed increasing charge transfer resistance for all anodes but was much more significant for the 0.10 mg_Ir_ cm^–2^ IrO*_x_* anode. These findings indicate that the composite anode improved stability due to mitigated increases in HFR and electrode resistance over time.

Moreover, degradation is often presented as a measure of the voltage loss rate in μV h^–1^ for long-term durability tests (i.e., constant current holds) or μV cycle^–1^ for ASTs. For example, the US DOE’s 2026 target degradation rate is 2.3 μV h^–1^, and their ultimate target is 2.0 μV h^–1^.^9^ While these values are based on continuous operation, they note that dynamic operation degradation must also be evaluated. The dashed lines in Figure 7a represent a linear fit of the stabilized degradation rate of cell voltage over the last ∼2000 cycles. The measured degradation rates were 1.5 and 5.9 μV cycle^–1^ for the 0.18 and 0.10 mg_Ir_ cm^–2^ IrO*_x_* anodes, respectively, indicating faster degradation for the lower Ir loading. This is consistent with the other recent work showing accelerated degradation for low Ir loadings with characteristically thin layers and high in-plane electrical resistances.^12,61^ The composite anode, however, showed a lower degradation rate for an Ir loading of 0.10 mg_Ir_ cm^–2^ with 3.1 μV cycle^–1^, indicating that the Pt black conductive additive helped slow down degradation during dynamic voltage cycling. In a recent investigation of catalyst type and AST procedure on low Ir loadings, Alia et al.^62^ reported a similar rate of 2.8 μV cycle^–1^ using the same AA IrO*_x_* catalyst, loading of 0.10 mg_Ir_ cm^–2^, and N115 membrane, although their protocol used current density cycling with 60 s cycles. We also note that other types of ASTs and long-term durability tests, such as chronopotentiometry or pressure cycling, would provide additional insight into the degradation mechanisms revealed by prolonged mechanical stress and are thus the subject of future work.

#### 3.3.2 Post-Mortem Imaging

**Figure 8:**
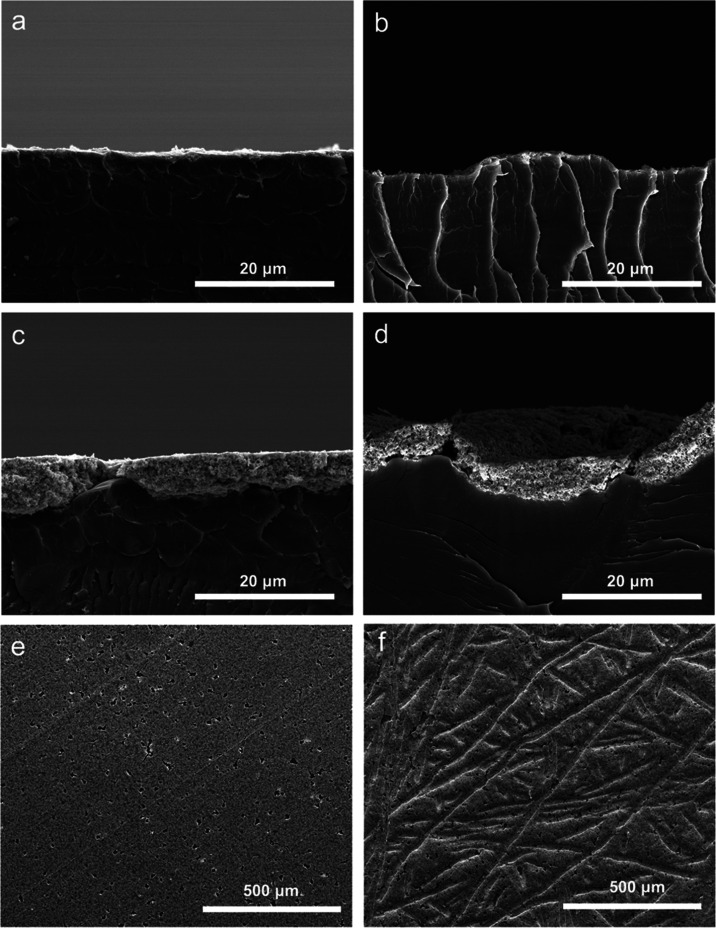
Cross-sectional SEM images of (a) pristine AA IrO*_x_* anode (0.11 mg_Ir_ cm^–2^), (b) postmortem AA IrO_x_ anode used in stability testing (0.10 mg_Ir_ cm^–2^), (c) pristine AA composite anode (0.12 mg_Ir_ cm^–2^ and 1.26 mg_Pt_ cm^–2^), and (d) postmortem AA composite anode used in stability testing (0.10 mg_Ir_ cm^–2^ and 1.05 mg_Pt_ cm^–2^). Surface SEM images of (e) pristine AA composite anode and (f) postmortem AA composite anode used in stability.

After ASTs, we took postmortem SEM images to investigate the possible physical degradation of the CLs. Cross-sectional images of AA IrO*_x_* anodes compare a pristine region in Figure 8a to a postmortem region from the stability-tested sample in Figure 8b. Similarly, a region of a pristine AA composite anode is shown in Figure 8c, and a postmortem region of the stability-tested composite anode is shown in Figure 8d. Surface SEM images of the pristine and postmortem composite anodes are also shown in Figure 8e,f, respectively. The indentations into the anode CL seen in both postmortem samples are due to compressed contact with the Ti fibers of the PTL and have been noted by others.^12,22,28^ While some local variations in CL thickness were seen in both the pristine composite anode (as discussed in the Section 3.1) and the postmortem composite anode, we do not see significant CL loss or overall thinning in the postmortem composite anode due to the AST. Furthermore, comparing the surface images of the composite anode before and after testing (Figure 8e,f) showed no noticeable deterioration or worsening of the small cracks and confirmed that the CL remained highly connected throughout testing, in agreement with the minimal increase in HFR. The electrochemical performance degradation results and postmortem imaging show that in this aggressive voltage cycling AST, the composite anode enhanced stability compared to an IrO*_x_* anode with the same low loading while maintaining its physical integrity.

### 3.4 Discussion of Implications and Limitations

Considering both outcomes of increased performance and enhanced stability, this composite anode design has several promising implications, yet it also has current limitations that allow for further understanding and improvement. The primary implication of a composite anode using a conductive additive to lower Ir loadings is that the approach is straightforward, diluting Ir via physically mixing the IrO*_x_* catalyst and conductive additive. Assuming that the desired catalyst and additive are commercially available products, fabrication of the composite anode is relatively simple and does not require complicated, time- or energy-consuming, or costly additional synthesis steps. This is a benefit of the composite anode design, as achieving scalable manufacturing of PEMWE components is another critical factor to consider alongside performance and durability, for example, for implementation into the roll-to-roll (R2R) method for production of PEM CLs.^16^ Similarly, increased conductivity and thickness of the composite anode design could eliminate the additional processing and costs incurred using an MPL for an improved PTL-CL interface. A final advantage of this composite anode design is that it could be a helpful laboratory diagnostic method for evaluating new catalyst materials. Its capability for robust performance with low Ir loadings can eliminate some of the cost barriers to experimenting with novel Ir catalyst structures since minimal catalyst is needed. Currently, limitations of the composite anode include small cracks and thickness variations in the CL, the relatively high Pt loading (>1 mg_Pt_ cm^–2^), the high cost of Pt compared to other cheaper and more abundant materials, and the need for longer-term durability assessments.

## 4 Conclusions

In this work, we show an initial implementation of a composite anode with a conductive additive to enable lower Ir loadings for PEMWE anodes. Specifically, we tested the use of Pt black as the conductive additive since the price of Pt is currently one-fifth that of Ir. We also suggest investigating other cheap and conductive materials for the PEMWE anode conductive additive. SEM and EDS images were used to confirm a good dispersion of IrO*_x_* particles throughout the Pt black while revealing areas of possible future work for fabrication optimization to reduce cracks and improve thickness uniformity. Sheet resistance measurements via a four-point probe showed that adding Pt black substantially lowered sheet resistance by over 3 orders of magnitude for Ir loadings lower than ∼0.2 mg_Ir_ cm^–2^. With the TKK IrO*_x_* catalyst, we achieved equal current density performance at 1.8 V between a composite anode with loadings of 0.10 mg_Ir_ cm^–2^ and 1.21 mg_Pt_ cm^–2^ and an IrO*_x_* anode with 1.88 mg_Ir_ cm^–2^. Assuming nominal prices of $5000 oz^–1^ of Ir and $1000 oz^–1^ of Pt, this translated to an 80% cost reduction of raw PGM materials with a 95% reduction in Ir loading. With the more stable and widely used AA IrO*_x_* catalyst, we achieved improved current density performance of 1.9 A cm^–2^ at 1.8 V for the composite anode with loadings of 0.10 mg_Ir_ cm^–2^ and 1.05 mg_Pt_ cm^–2^ while also showing that the composite anode enhanced stability throughout 10.8k AST cycles compared to a standard IrO*_x_* anode with the same loading of 0.10 mg_Ir_ cm^–2^. In summary, initial success has been demonstrated using a composite anode approach with a conductive additive to lower Ir loadings while maintaining high current density performance at moderate cell voltages. Still, there is room for further cost improvement by optimizing the composite anode design with Pt black conductive additive and investigating other lower-cost, highly conductive materials as the additive for a PEMWE composite anode.

## Supporting Information Available

Additional SEM surface and cross-sectional images, EDS mapping images for composite anodes, EIS data, stability cycling profile and time-series data, and AST polarization curves (PDF)
