# Supplementary material for: Composite Anode for PEM Water Electrolyzers: Lowering Iridium Loadings and Reducing Material Costs with a Conductive Additive
Source: ACS Appl Energy Mater. 2024 Sep 6;7(18):8124–35. doi: 10.1021/acsaem.4c01866 (PMC11423428; doi:10.1021/acsaem.4c01866)
Supplement: Supplementary file 1 — ae4c01866_si_001.pdf [file ae4c01866_si_001.pdf]

Supporting Information

Composite anode for PEM water electrolyzers: Lowering iridium loadings and reducing material costs with a conductive additive

Kara J. Ferner[a] and Shawn Litster[a,*]

[a]Department of Mechanical Engineering, Carnegie Mellon University, 5000 Forbes Avenue, Pittsburgh, PA 15213, USA

[*]Corresponding Author Email Address: litster@andrew.cmu.edu

Supplementary figures

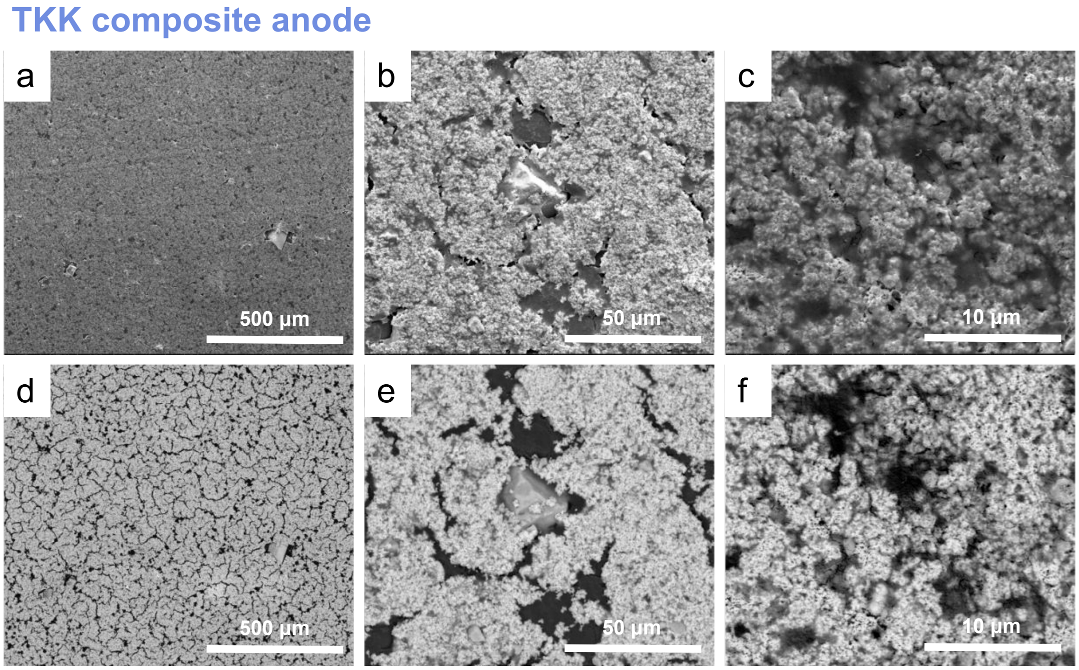

Figure S1. TKK composite anode surface SEM images. (a-c) Secondary electron images of increasing magnification and (d-f) back scattered electron images of increasing magnification.

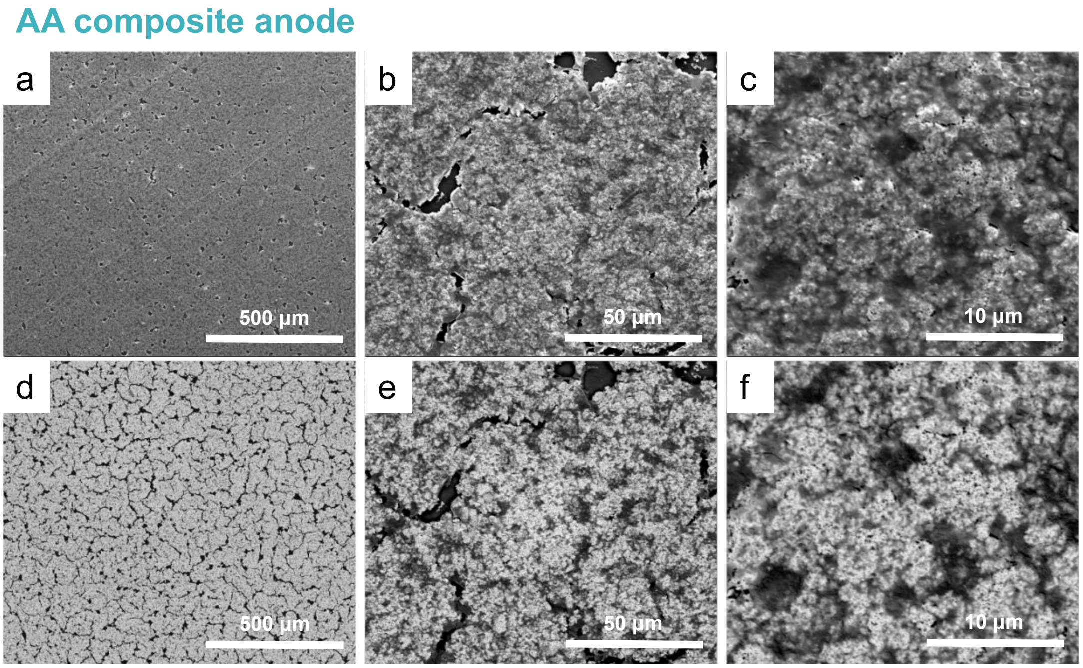

Figure S2. AA composite anode surface SEM images. (a-c) Secondary electron images of increasing magnification and (d-f) back scattered electron images of increasing magnification.

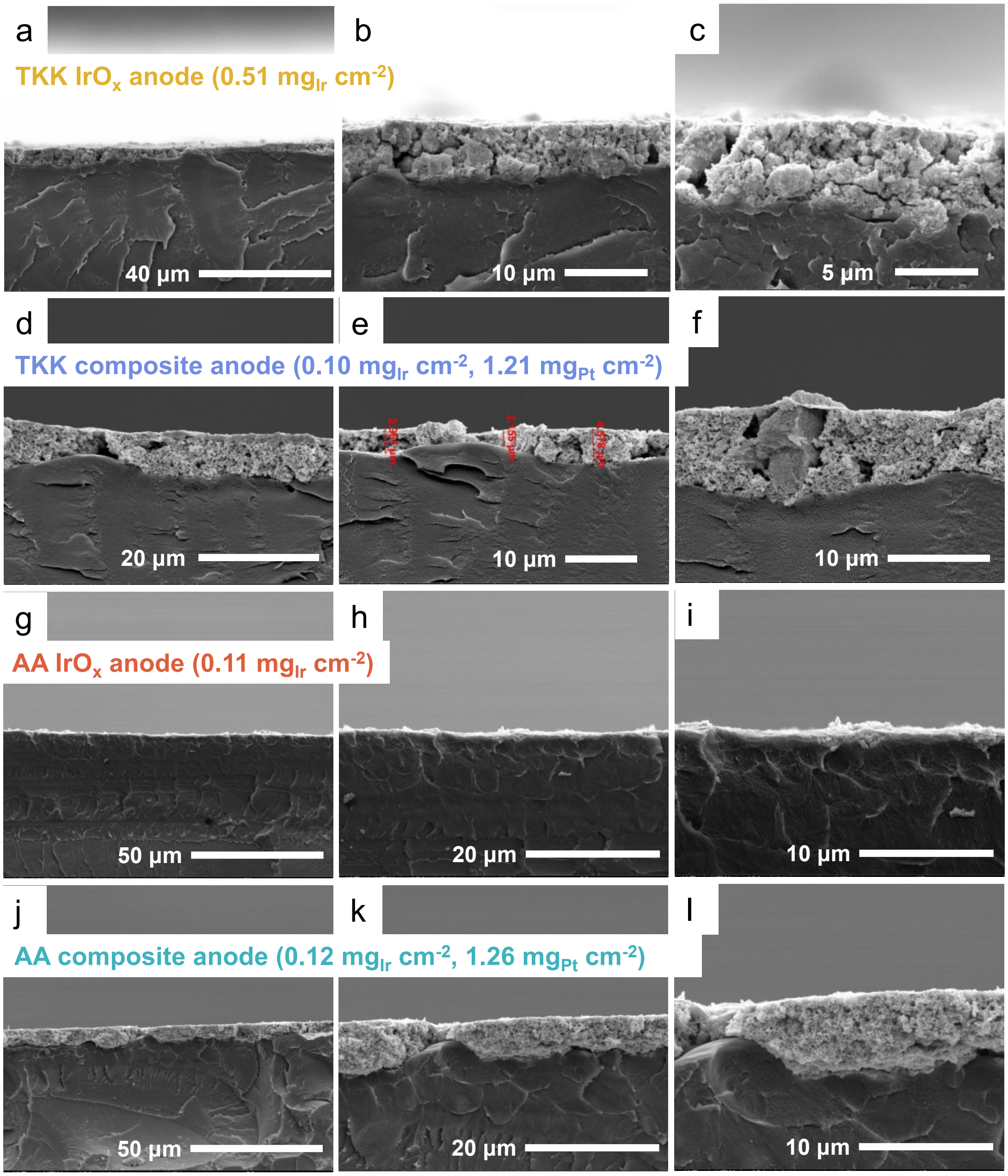

Figure S3. Cross-sectional SEM images of the different types of anodes in this study (on Nafion™□ 115 membrane). (a-c) TKK IrO_x anode with a loading of 0.51 mg_{Ir} cm^{-2}, (d-f) TKK composite anode with a loading of 0.10 mg_{Ir} cm^{-2} and 1.21 mg_{Pt} cm^{-2}, (g-i) AA IrO_x anode with a loading of 0.11 mg_{Ir} cm^{-2}, and (j-k) AA composite anode with a loading of 0.12 mg_{Ir} cm^{-2} and 1.26 mg_{Pt} cm^{-2}.

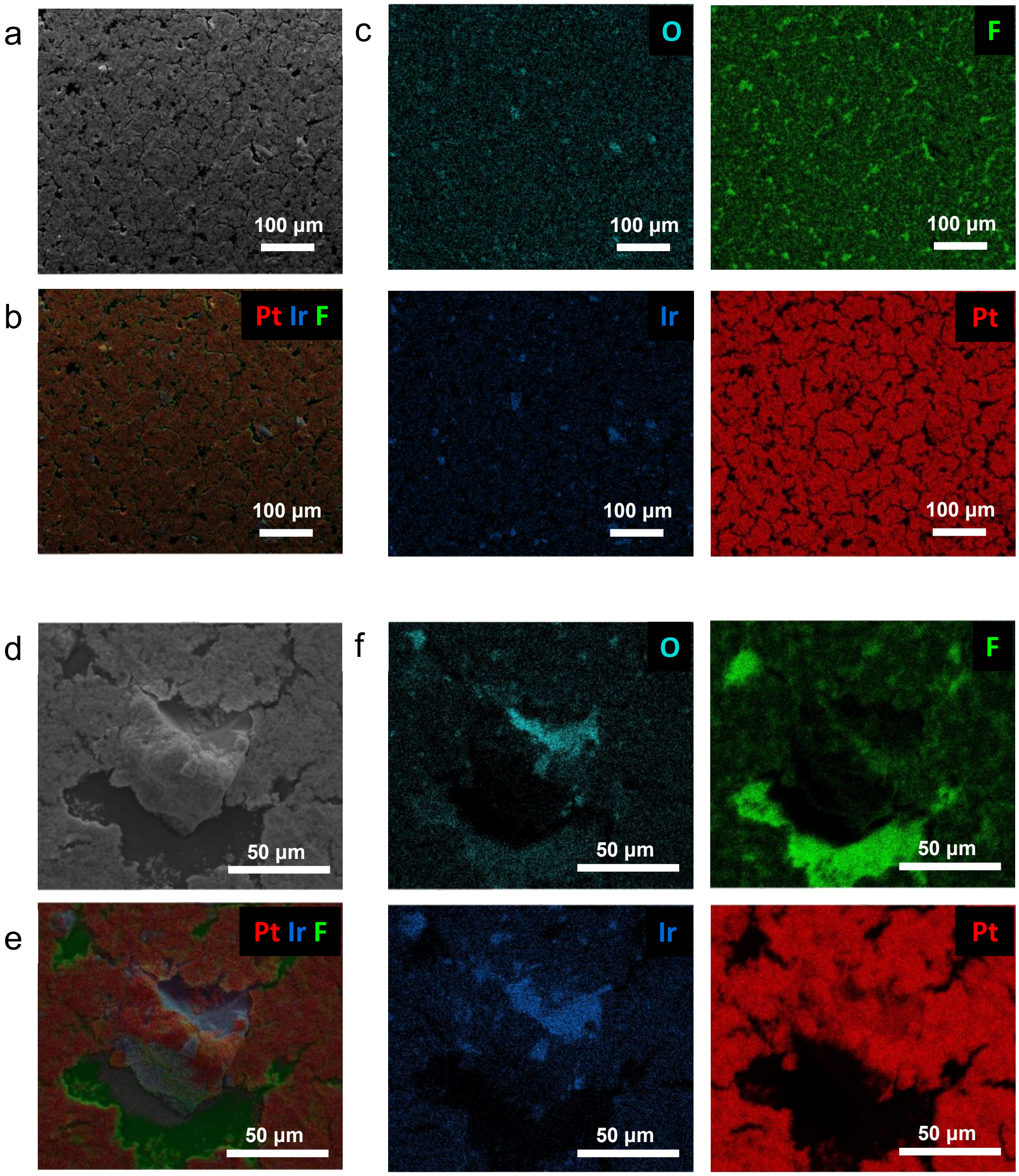

Figure S4. TKK composite anode surface SEM images with EDS mapping. Region of a larger field of view, lower magnification with (a) secondary electron image, (b) elemental map overlay, and (c) individual elemental maps. Region of a smaller field of view, higher magnification with (a) secondary electron image, (b) elemental map overlay, and (c) individual elemental maps.

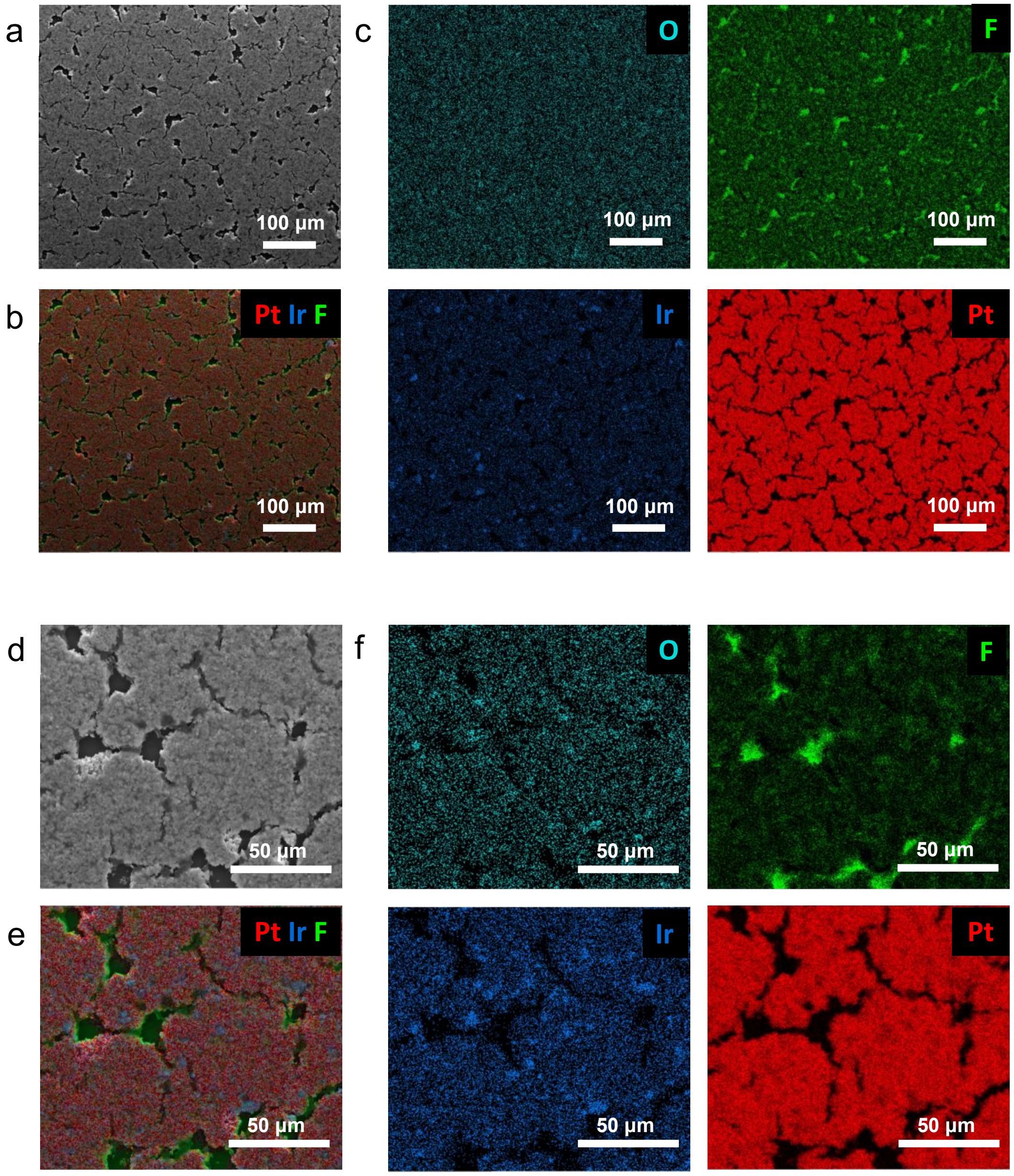

Figure S5. AA composite anode surface SEM images with EDS mapping. Region of a larger field of view, lower magnification with (a) secondary electron image, (b) elemental map overlay, and (c) individual elemental maps. Region of a smaller field of view, higher magnification with (a) secondary electron image, (b) elemental map overlay, and (c) individual elemental maps.

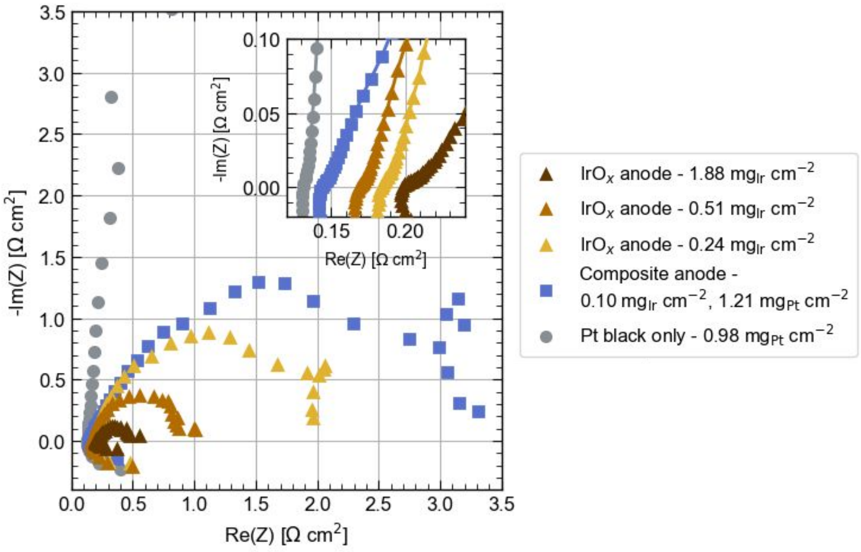

Figure S6. EIS data at 1.4 V on Nyquist plot for TKK IrO_x anodes, TKK composite anode, and anode with Pt black only. The diameter of the characteristic high-to-low frequency semicircle in this data represents the charge transfer resistance associated with each electrode. Charge transfer resistance increases with lower Ir loading due to the reduced catalyst surface area. The increase in charge transfer resistance seen in the TKK IrO_x is due to the significantly lower Ir loading used rather than caused by the composite anode design itself. The HFR values of the three IrO_x anodes are slightly higher than the composite anode and Pt black anode, possibly due to the slightly thicker membrane thickness (147 µm) used for those three cells compared to the other cells (127 µm). These thick membranes needed for PEMWEs (as opposed to < 50 µm for commercial fuel cells) are the most significant contribution to the HFR value, so improvements in HFR due to catalyst layer changes are comparatively smaller.

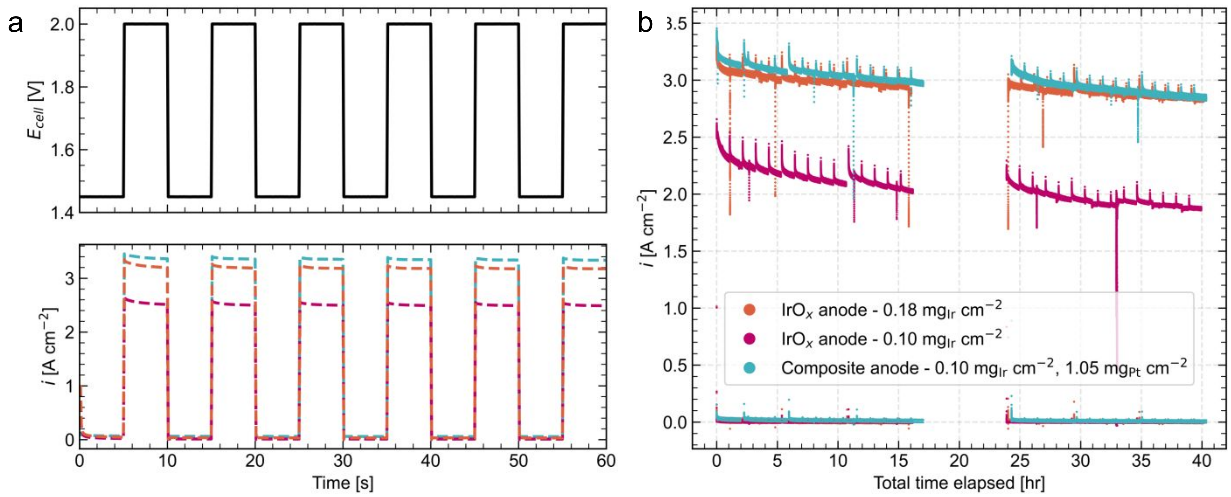

Figure S7. (a) Potentiostatic cycling profile of the modified AST test: 10-second cycles between 1.45 V and 2 V. (b) Full time-series data of the current density response during the cycling.

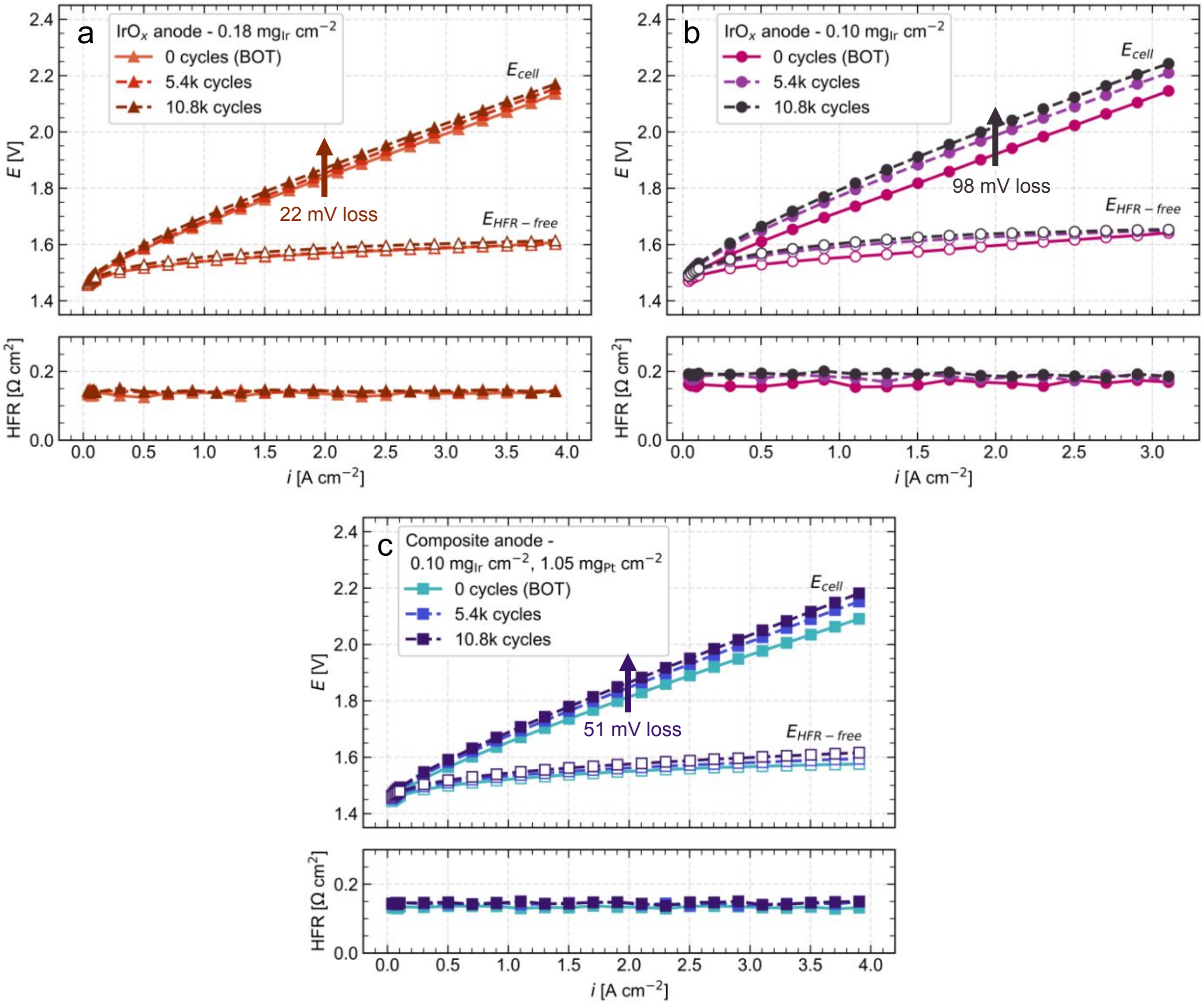

Figure S8. Polarization curves at BOT, after 5.4k cycles, and after 10.8k cycles of AST stability testing for (a) 0.18 mg_{Ir} cm^{-2} IrO_x anode, (b) 0.10 mg_{Ir} cm^{-2} IrO_x anode, and (c) composite anode with 0.10 mg_{Ir} cm^{-2} and 1.05 mg_{Pt} cm^{-2}.

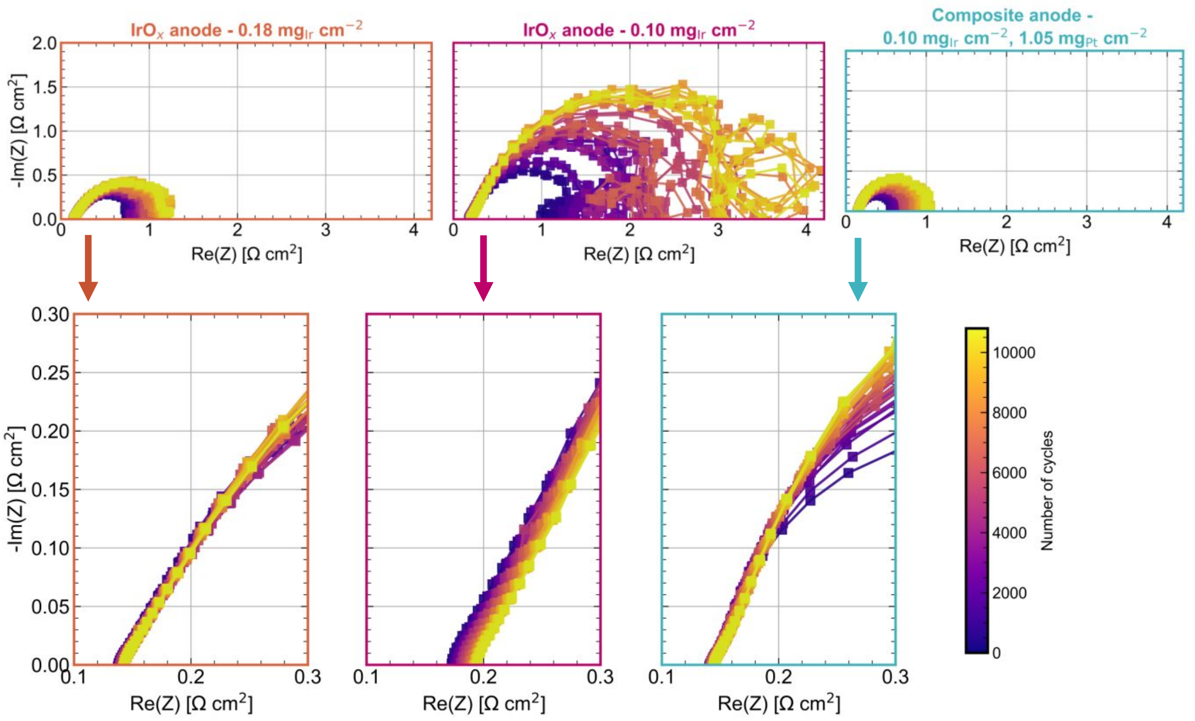

Figure S9. EIS data for the three AA anodes throughout the AST. The color bar shows the change in EIS data vs. number of cycles of completed AST, where a new spectrum was taken every hour of testing (after every 360 cycles and followed by the 1-minute 2 A cm^{-2} hold), and was done at a voltage of 1.45 V, frequencies from 100 kHz to 100 mHz, and amplitude of 5 mV. The top row of Nyquist plots shows the full range on identically scaled axes for all three samples, clearly showing the much larger charge transfer resistance for the 0.10 mg$_{Ir}$ cm^{-2} IrO_x anode compared to the 0.18 mg$_{Ir}$ cm^{-2} IrO_x and the composite anode. The bottom row of Nyquist plots shows a magnified range on identically scaled axes for the high-frequency region, showing the HFR increase over the AST was also more significant for the 0.10 mg$_{Ir}$ cm^{-2} IrO_x anode comparatively.
